# Dyspepsia

**Published:** 1853-06

**Authors:** 


*Dyspepsia.*—Dr. Bennet (Ed. Monthly Journal, Feb. 1853), in a lecture on Dyspepsia, after insisting on the necessity of seeing that there is no excess in eating and drinking, that the food is properly masticated, and that proper rest is taken after food, remarks that the sense of load or weight is best relieved by acids, especially the hydrochloric. Acid eructations and cardialgia are best relieved by alkalies and bitter tonics. In cases in which fatty matters do not appear to be digested, liq. potassæ is recommended. When the flow of bile appears deficient, mild mercurials and rhubarb is the best treatment.

